# Piezoresistive Properties of Natural Hydraulic Lime Binary Pastes with Incorporated Carbon-Based Nanomaterials under Cyclic Compressive Loadings

**DOI:** 10.3390/nano12203695

**Published:** 2022-10-21

**Authors:** Angeliki-Eirini Dimou, Zoi S. Metaxa, Stavros K. Kourkoulis, Nikolaos D. Alexopoulos

**Affiliations:** 1 Research Unit of Advanced Materials, Department of Financial Engineering, School of Engineering, University of the Aegean, 82132 Chios, Greece; 2 Department of Chemistry, International Hellenic University, St. Luke, 65404 Kavala, Greece; 3 Laboratory of Testing and Materials, Department of Mechanics, National Technical University of Athens, 15780 Athens, Greece

**Keywords:** natural hydraulic lime, metakaolin, carbon nanomaterials, carboxylated graphene, graphene oxide, reduced graphene oxide, carboxylated CNTs, sulfonated CNTs

## Abstract

Natural Hydraulic Limes (NHL) are extensively used for the restoration of Monuments of Cultural Heritage, often combined with pozzolanic materials, such as natural pozzolans and metakaolin etc. In the present study, five (5) different cases of binary lime-based pastes composed of a specific type of NHL (NHL5) and metakaolin as pozzolanic addition were examined, that were reinforced with carbon nanostructures, namely graphene and carbon nanotubes. For the first time in restoration mortars, the incorporation of carbon nanostructures was investigated, aiming to produce materials with adequate piezoresistive response, so that they have the potential to be exploited for in situ structural health monitoring. The compressive strength, flexural strength, electrical resistance and piezoresistive response of the composite pastes was examined. The results showed that all modified carbon nanostructures lead to a significant reduction in electrical resistance. The pastes reinforced with 2D nanostructures (graphene family) displayed up to 30% increase in compressive strength and the pastes reinforced with 1D nanostructures (carbon nanotubes) displayed enhanced flexural strength (up to 100% increase). Piezoresistivity was attained for almost all investigated pastes, nevertheless the graphene oxide (GO) was considered as optimal reinforcement as the sensing ability of such pastes was found to be almost proportional to the applied compressive load level.

## 1. Introduction

The structural integrity of historic and traditional buildings can be affected by natural weathering and incompatible restoration interventions. Traditionally, hydrated lime binders have been used for restoration applications. However, they were gradually replaced by hydraulic materials and by the beginning of the 20th century, mainly by cement [1]. During the previous decades, the extensive use of cement-based mortars for conservation activities was often criticized. According to Fang et al. [2] they resulted in accelerating the deterioration due to microstructure incompatibility and formation of soluble salts. In recent years, the negative effects due to the inappropriate use of incompatible cement–based mortars have shifted the scientific interest once more to the use of lime-based restoration materials. To this end, the development of compatible binder matrices for restoration mortars is always a crucial point for designing effective and sustainable interventions [3].

Natural hydraulic lime is obtained from the calcination and slaking of limestone naturally containing clay impurities [4]. NHL mortars can harden both in standard air-dry conditions and in high humidity environments, through the various reactions which take place during hardening (hydration and carbonation), providing an additional advantage regarding their use. In recent years, the study of high hydraulicity NHL mortars has been in the focus of many researchers, as it is considered a compatible, eco-friendly binding material [4].

In many cases, lime, either hydrated or hydraulic, is not used alone in restoration mortars. According to [1,5], there are records and archaeological sites which prove that ancient civilizations used limes with pozzolans for the preparation of mortars with hydraulic characteristics namely to be into contact with water, which contributed to the development of limes with hydraulic properties. Grilo et al. [1] used metakaolin (MK) as pozzolanic addition to improve the mechanical resistances of the NHL mortars. They found that the partial substitution of lime by metakaolin can be useful to adjust the mortars characteristics for different types of supports and applications sites.

Due to its allotropic characteristics, carbon forms compounds that have completely different properties depending on the arrangement of the adjacent carbon atoms. Carbon nanostructures (fullerenes, nanotubes, nanodiamonds etc.) are among the most promising in nanotechnology because they can be used in different fields. Composites materials derived from carbon-based nanostructures (CBNs) exhibit advanced physical and chemical properties, an asset that can be useful for many different applications, e.g., new engineering products, biomedical applications [6].

One category of CBNs, which has been gaining scientific interest in the last years is graphene and its derivatives. Graphene (G) is a nanomaterial arranged in a two-dimensional layer of carbon atoms with sp^2^ hybridization that are connected in a hexagonal lattice structure. Graphene has a remarkable band structure due to its crystalline structure [6]. Carbon nanotubes (CNTs) are seamless nanoscale tubes structurally built from rolled monolayer or multilayer grapheme sheets, which possess covalent sp^2^-hybrid bonds between individual carbon atoms. Mechanically, the exciting features of carbon nanotubes (CNTs), such as high elastic modulus, high thermal and electrical conductivities, robustness, and nanoscopic surface properties make them attractive candidates for building materials. CNTs can arrest propagating cracks due to their high aspect ratio and large specific surface area, e.g., [7,8].

Regarding new engineering products and composite materials, the most challenging issue is the difficulty in dispersing the nanostructures. The van der Waals forces among the carbon nanoparticles cause intense agglomeration. This phenomenon leads to the reduction of the specific surface area of the nanoparticles, as well as to an inhomogeneous dispersion of the nanoparticles in the composite material [9]. Zhao et al. [10] reported that the lack of homogeneity results in the creation of weak zones in the final product. Controlling the dispersion is a crucial factor since it has been well established that the mechanical behavior of CNTs and graphene is also strongly influenced by the van der Waals forces [11].

To this end, it is extremely important to break down any-formed agglomerates and to efficiently disperse the nanoparticles to form a stable system. The most common method to achieve this goal is through the application of ultrasonic energy. Another way to enhance the dispersion of CBNs in different matrices is the surface functionalization of the nanostructures [12]. Covalent functionalization is an important means to form covalent linkage between the main structural units of G or CNTs with functional groups. Covalent interactions on the surfaces are favored due to the presence of hydroxyl, epoxy, and carboxylic groups; considered the best surface treatments for the functional group conversion [12,13].

The present study focuses on the incorporation of CBNs into restoration materials that usually contain no cement in the mixture. So far, CNTs, G, and their derivatives have successfully been incorporated in cement-based materials to produce composite materials with enhanced mechanical properties, as well as self-sensing properties due to their piezoresistive characteristics. Liu et al. [14] studied the effects of graphene and graphene oxide nanoplatelets in mechanical properties, electrical resistance and piezoresistive reactions of cementitious mortars. They produced mortars with varying concentrations of nanomaterials (even more than 10%). They found that electrical resistance decreases with an increase in conductive nanoparticles’ concentration. However, the compressive strength of the mortars increased when the concentration of nanoplatelets remained less than 0.2 wt.%. Regarding the piezoresistive characteristics, they found that cement/graphene nanoplatelets composites possess self-sensing characteristics. Guo et al. [15] studied the effect of graphene oxide (GO) in a cementitious matrix. In contrast to the study of Liu et al. [14], who reported that graphene oxide nanoplatelets are not a suitable candidate for smart sensors, Guo et al. found that the incorporation of GO in the cement paste enhances conductive and piezoresistive properties, even at very small concentrations of approximate 0.05 wt.%.

Yu and Kwon [16] investigated the piezoresistive characteristics of cement composites with incorporated multi-walled CNTs (MWCNTs). Two different concentrations of MWCNTs (0.06 and 0.1 wt.%) were examined and it was found that the piezoresistive response of both composites was about 9% and 10 to 11% at loading levels of 5.2 MPa and 8.6 MPa, respectively. Their experimental results showed that the electrical resistance of the composites changes under varying compressive stress level, indicating the potential of using the CNT/cement composite as a stress sensor for civil structures [16]. Cwirzen et al. [17] studied the effect of MWCNTs on the mechanical properties of cement pastes. Different types of MWCNTs were used, like plain MWCNTs as well as functionalized MWCNTs with carboxyl groups. In both cases, compressive strength increased in comparison to pure cement. Nevertheless, acidic treatment led to the production of more stable and homogenous aqueous dispersions, so the highest increase in the compressive strength was nearly 50% in cement paste when the functionalized CNTs (at only 0.045 wt.%) were added [17].

Manzur and Yazdani [18] studied the incorporation of surface treated MWCNTs in cement composites. The compressive and flexural strength of the composites were examined at different dosage rates of nanostructure, water/cement ratios and plasticizer amounts (as surfactant). They found that concentrations of MWCNTs ranging from 0.1 to 0.3 wt.% led to a 15% increase in compressive strength and the concentration of 0.1 wt.% MWCNTs led to 19.5% increase in flexural strength (both at 28 days of age) [18]. Li et al. [19] also studied surface treated MWCNTs, treated with the strong acids H_2_SO_4_ and HNO_3_. Their results showed that the treated MWCNTs improved the flexural strength, compressive strength, and failure strain of the cement matrix composites, as the nanoparticles fine the pore size distribution and decrease porosity [19].

Although literature regarding cementitious nanocomposites is very extended, literature regarding NHL nanocomposites for the restoration of Moments of Cultural Heritage is extremely limited. For example, Faria et al. [20] were the first to incorporate GO in an NHL matrix, driven by the demand to enhance the properties of the traditional materials. Likewise, Barberro-Barrera and Medina [21] studied NHL composites with incorporated graphite (carbon material) and polymeric fibers, while Luo et al. [22] incorporated a totally different nanomaterial in NHL, namely nano-SiO_2_. None of these studies focuses on piezoresistive characteristics of the composite NHL materials. The aim of the present investigation is the development of compatible nanocomposite materials for restoration applications with self-sensing properties and piezoresistive characteristics. For that reason, a binary lime-based (NHL/MK) paste will be investigated, in which five (5) different modified carbon nanostructures will be incorporated. Two cases refer to modified MWCNTs and three cases to modified graphene nanostructures.

## 2. Materials and Methods

**Figure 1:**
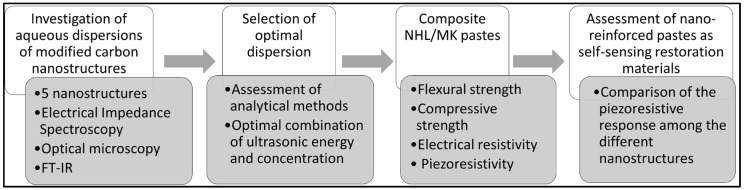
Schematic experimental process of the present study.

This section describes the experimental methodology followed in the present study and can be summarized as follows: (1) aqueous dispersions of five different carbon nanostructures were examined via analytical methods and electrical impedance spectroscopy to find the optimal combination of nano-reinforcement concentration and ultrasonic energy; (2) NHL/metakaolin composite pastes were produced with the “optimal” dispersions derived from the 1st step; (3) mechanical, electrical, and piezoresistive tests under cyclic loading were performed on the composite pastes. The experimental methodology is described in the following diagram (Figure 1):

### 2.1. Materials

#### 2.1.1. Aqueous Dispersions of Carbon Nanostructures

Five different modified carbon nanostructures were examined in this study, three modified graphene nanostructures and two modified carbon nanotubes: (i) graphene oxide (GO), (ii) carboxylated graphene (GCOOH), (iii) reduced graphene oxide (rGO), (iv) carboxylated multiwall carbon nanotubes—MWCNTs (MWCNTsCOOH), (v) sulfonated MWCNTs (MWCNTsOSO_3_H). Several nanostructures were produced in laboratory scale at the University of Ioannina, Department of Materials Science & Engineering (Prof. Karakasides and Prof. Gournis), under the framework of the Greek National research project “AKEISTHAI”. Chemical functionalization is very important, when stability and the mechanical properties of the modified graphene are required [23]. Likewise, modification of MWCNTs improves their compatibility and stability, enhancing the potential of MWCNTs in sensing applications, e.g., [24]. For each nanostructure, aqueous dispersions were produced to find the optimal combination of ultrasonic energy and nano-reinforcement concentration (as wt.% of the binder), as described in previous works of the authors, e.g., [25,26]. The dispersion medium in the present investigation was bottled water (Epirotic Bottling Industry S.A.—VIKOS S.A.).

**Figure 2:**
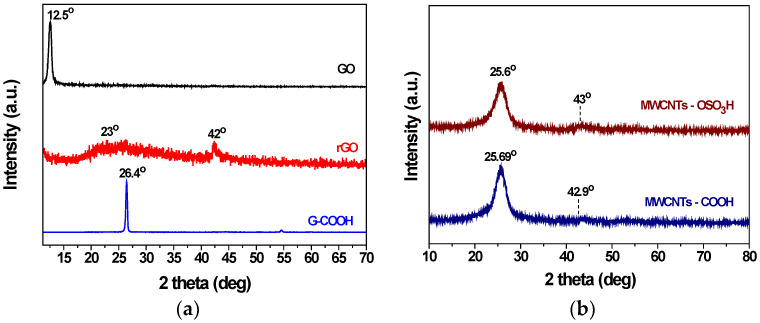
XRD patterns of (**a**) Graphene Oxide (GO), reduced Graphene Oxide (rGO) and Carboxylated Graphite (G-COOH) and of (**b**) MWCNTs carboxylated and sulfonated, respectively.

XRD patterns of GO, rGO and G-COOH are shown in Figure 2a. The XRD spectra were collected on a D8 Advance (Bruker, Billerica, MA, U.S.A.) diffractometer by using Cu Ka radiation (40 kV, 40 mA) and a secondary beam graphite monochromator. The diffraction peak after the graphite’s oxidation is observed at 2θ = 12.26° which corresponds to d001—spacing of 7.4 Å indicating the successful oxidation of graphite powder to graphene oxide (GO). As shown in the rGO XRD pattern the peak of the reduced GO (002) appeared at 2θ = 23° which is the result of the reduction procedure. In the X-ray diffraction pattern of G-COOH a high intensity peak is shown at 2θ = 26.44° which is attributed to the graphitic structure (002). Figure 2b shows the XRD patterns of carboxylated multi-walled carbon nanotubes (MWCNTs–COOH), and sulfonated multi-walled carbon nanotubes (MWCNTs–OSO_3_H). In the X-ray pattern of MWCNTs-COOH occurrence of (002) is observed, at 2θ = 26.89°. The presence of (100) diffraction is also observed at 2θ = 42.91°. Both diffractions are attributed to the structure of MWCNTs-COOH. The XRD pattern of MWCNTs–OSO_3_H shows that the graphitic conformation is preserved and is the result of the main diffraction peak (002) at 2θ = 25.79°.

**Figure 3:**
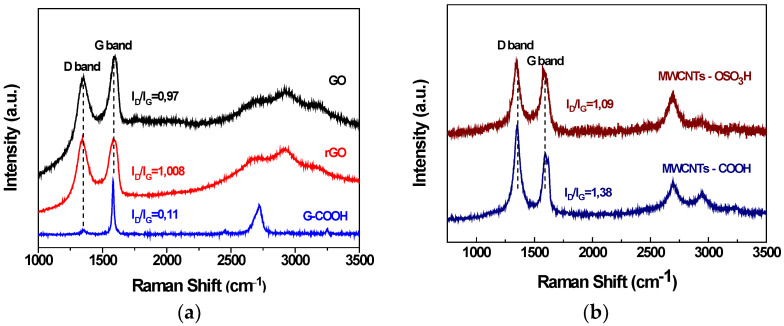
(**a**) Raman spectra of GO, rGO, and G-COOH and (**b**) Raman spectra of MWCNTs-COOH and MWCNTs-OSO_3_H.

The Raman spectrum of the graphene oxide is shown in Figure 3a and exhibits the characteristics bands. Raman spectra were recorded with a micro-Raman Renishaw RM1000 system (Renishaw, Kingswood, UK). The D band is observed at 1353 cm^−1^ and is attributed to sp^3^ domains by the creation of lattice defects and distortions during the oxidation of the graphite powder. The first order G band at 1597 cm^−1^ is assigned to sp^2^ hybridized carbon atoms of the graphitic lattice. The ratio (I_D_/I_G_) of D and G bands indicates the formation of the oxygen functional groups during the oxidation and was found to be 0.84. During the reduction, the ratio of D and G bands’ is slightly increased because of the removal of oxygen containing functional groups on the surface as well as at the edges of graphene oxide’s nanosheets. In the spectrum of G-COOH the ratio of bands (I_D_/I_G_) is about 0.11 which is attributed to the existence of -COOH groups in the graphite. The increased band intensity at 1580 cm^−1^ indicates the sp^3^ hybridization of the carbon atoms in the graphite lattice. The Raman spectrum of carboxylated carbon nanotubes (MWCNTs-COOH) is shown in Figure 3b. It is observed that I_D_/I_G_ bands’ ratio takes the value of 1.38 attributed to the existence of -COOH groups. Raman spectrum of MWCNTs-OSO_3_H is also shown in Figure 3b and features the I_D_/I_G_ ratio which acquires the value of 1.09 due to the presence of sulfonic groups.

#### 2.1.2. Lime-Based Pastes

The hydraulic pastes were prepared by mixing commercial Natural Hydraulic Lime NHL5 (St. Astier Natural Hydraulic Limes, London, UK) and Metakaolin (MK) Metacal 3000 (CALTRA Nederland B.V., Mijdrecht, The Netherlands) with weight contents 80 wt.% and 20 wt.%, respectively. Hydraulic limes are often used in restoration applications [4], while pozzolanic materials are often added in cement and lime materials as admixtures in weight percentages up to about 30%. Specific characteristics about the binder materials can be seen in Table 1, all obtained from the respective technical data sheets.

The water to binder (W/B) ratio was set at 0.55, based on previous research article of the authors [27]. Based on the evaluation of the aqueous dispersions (more details can be found in the Results section), one “optimal” combination of ultrasonic energy and concentration was selected to produce composite pastes for each, different carbon nanostructure. Thus, six (6) cases of pastes were examined in total in the present investigation, consisting by one (1) for the plain paste (no nanostructures reinforcement) and five (5) different pastes with different incorporated modified nanostructures each. The composition of the pastes can be seen in Table 2.

### 2.2. Methods

#### 2.2.1. Aqueous Dispersions Preparation and Evaluation

**Figure 4:**
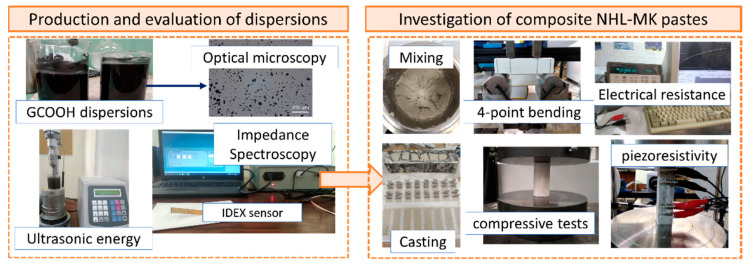
Summary of the experimental methods performed in the present study.

The methodological approach of the present research can be seen schematically in the following diagram (Figure 4). For the preparation of the aqueous dispersions, 100 mL water and each nanostructure were weighed and placed in a beaker and were slightly hand-stirred. Dispersion was accomplished with the use of a tip ultrasonicator (VCX-500—energy generator, CV-334 model—nozzle by SONICS & MATERIALS^®^, Newtown, CT, USA). A temperature sensor was also used to ensure that the temperature would not exceed 50 °C. The examined concentrations of the dispersions were 0.05, 0.10, 0.15 and 0.20 wt.% of the binder, while ultrasonic energy amounts reached up to 100 kJ (maximum applied). Next, an appropriate combination of ultrasonic energy and concentration was selected for each nanostructure to ensure the production of homogenous aqueous dispersions. The selected dispersions were then incorporated into the binder matrix as mixing water to produce the composite materials.

The evaluation of the aqueous dispersions was performed with a methodology developed in [25] that exploits Electrical Impedance Spectroscopy (EIS) measurements. EIS was performed during the ultrasonication process using a Dielectric Thermal Analysis System DETA-SCOPE^®^ (ADVISE, Chios, Greece). The hardware setup was connected to an interdigital dielectric sensor IDEX^®^ (Netzsch, Ahlden, Deutschland), with dimensions 10 mm in width and 30 mm in length. All impedance scans were performed during the ultrasonication process at different accumulated ultrasonic energy values (namely 3, 12, 30, 65 and 100 kJ) and were recorded at a personal computer. The specific impedance measuring device was selected due to its potential to be used for in situ applications, as it is highly portable and provides high-precision measurements.

Although EIS was applied to characterize the aqueous dispersions of carbon nanostructures, other instrumental methods were also exploited to investigate the above dispersions and for the same ultrasonic energy values. Light optical microscopy was also involved for the evaluation, where a Leica DM-RX microscope was used to examine the samples subjected at various ultrasonic energy values. The micrographs obtained from the optical microscopy were then examined regarding the nanoparticle size. The progression of specific peaks was studied to investigate whether the high accumulated ultrasonic energy values can destroy the already formed, specific functional groups.

#### 2.2.2. Production of the Pastes

Mixing was performed using a standard 5 L mixer made by TECHNOTEST^®^, Quezon, Philippines. NHL and MK were placed in the mixing bowl and then the aqueous dispersion (prepared with the carbon nanostructures from the previous step) was added. At first, the mixer was set to rotate at low speed for 30 s. Next, its function was paused for 15 s to scrape down the paste that remained on the sides of the bowl. Finally, the mixer was set to rotate at medium speed for 60 s. Two types of molds were used to cast the previously described mixtures: prismatic molds (80 mm × 20 mm × 20 mm) and cylindrical molds with dimensions 30 mm in diameter and 60 mm in height. After mixing, the pastes were cast in the molds and were left to cure for 24 h at approximate 20 °C. Next, the specimens were demolded and placed in a water tank with calcium hydroxide for curing (28 days).

#### 2.2.3. Electrical and Mechanical Characterization of the Specimens

Electrical resistance testing was performed on similar prismatic specimens as above with the four-probe method. The A 34970A Data Logger (Keysight Technologies, El Segundo, CA, USA) was used for the experiments. Before the tests and for the removal of the moisture from the specimens, they were dried in an oven for 3 days at 60 °C.

The cylindrical specimens were used for the compression mechanical tests, where the mechanical tests were performed at a 300 kN Instron SATEC^®^, Jerusalem, Israel, loading frame. The crosshead displacement rate was kept constant during the execution of the test and was equal to 300 μm/min. The prismatic specimens were used for the 4-point testing of the pastes. The flexural mechanical tests were performed at a 10 kN MTS Insight^®^ loading frame. The crosshead displacement rate was kept constant and equal to 0.001 mm/s. All experimental data were continuously recorded in a P/C.

Piezoresistive response under cyclic compressive loading was investigated for all five different pastes. It must be noted that the reference paste (without any carbon nanostructures reinforcement) did not exhibit piezoresistive characteristics. The 10 kN Insight testing machine and the 34970A Data Logger for the electrical measurements were used for these kinds of experiments. The compression cyclic tests were performed with a constant load amplitude, with the starting/returning loading value being zero, while the peak load value was selected based on the results of the monotonic compressive tests. Three different cases were examined in the present investigation, that corresponded to different peak load values of the cyclic tests. The peak load values were selected to be approximate at 10%, 20% and 50% of the compressive strength, respectively. The selection of the different maximum loads aimed to investigate the capability of the nano-reinforced pastes to exhibit piezoresistive characteristics under different loading spectra that induces different types of internal damage on the matrix. In all cases, the electrical resistance was in situ recorded during 15 loading–unloading testing cycles.

## 3. Results and Discussion

### 3.1. Evaluation of Dispersions

**Figure 5:**
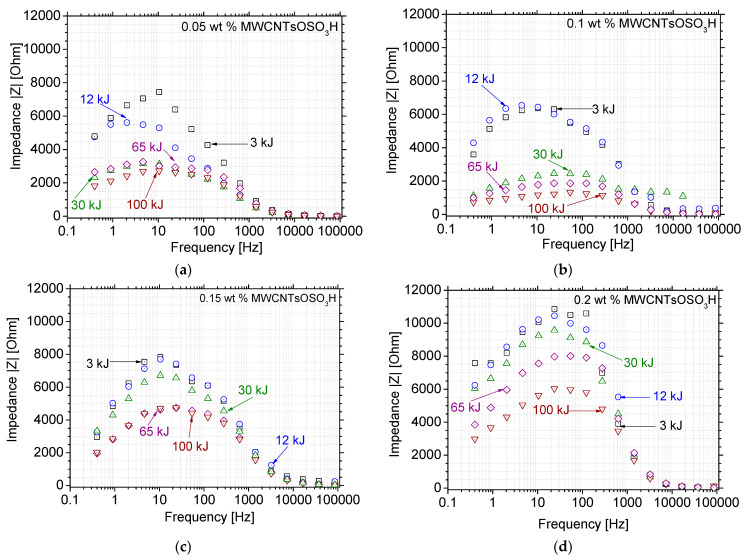
Bode plots of MWCNTsOSO_3_H aqueous dispersions at different ultrasonic energy levels and concentrations of (**a**) 0.05, (**b**) 0.10, (**c**) 0.15 and (**d**) 0.20 wt.%, respectively.

**Figure 6:**
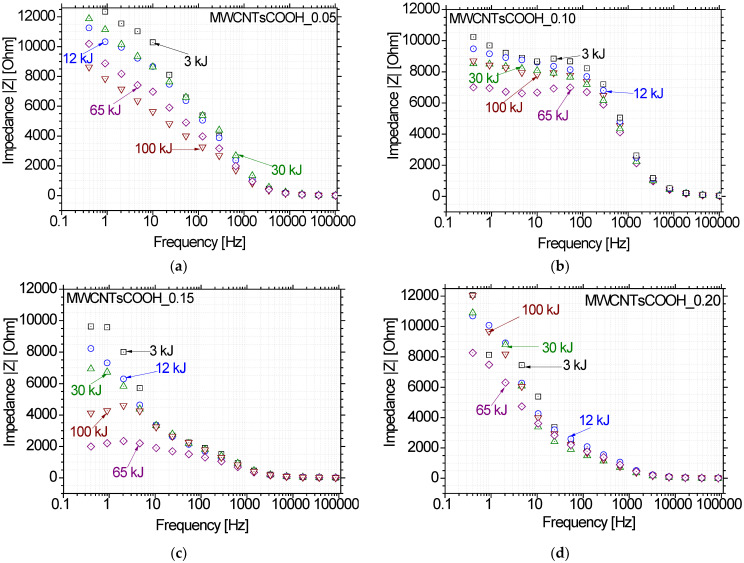
Bode plots of MWCNTsCOOH aqueous dispersions at different ultrasonic energy levels and concentrations of (**a**) 0.05, (**b**) 0.10, (**c**) 0.15 and (**d**) 0.20 wt.%, respectively.

The impedance spectra of the dispersions were interpreted via their respective Bode Plots. The respective Bode plots for all graphene-based nanostructures can be seen in [25,26], while similar approach was followed for the MWCNTs nanostructures. Figure 5 and Figure 6 display the Bode plots for the two different types of MWCNTs dispersion investigations and for four different concentrations per type. Figure 5 shows the respective results for the sulfonated MWCNTs. The Bode plots show that an increase in ultrasonic energy leads to a reduction of impedance modulus |Z|. This behavior is different in comparison to graphene nanostructures, where an inversion is noted after a certain amount of ultrasonic energy [25]. This can be attributed to the structural differences among the nanostructures. In contrast to other modifications, treatment with H_2_SO_4_ leads to a hydrophilic material with high acidity [28]. Additionally, sulfonation leads to a decrease in the MWCNTs surface area and an alteration of the surface properties [29].

The 0.10 wt.% dispersions display the lowest impedance modulus |Z| at high ultrasonic energy values and higher frequencies. Due to polarization phenomena that may affect the measurements, the impedance values at very low frequencies (i.e., *f* < 100 Hz) were not taken into consideration. The highest concentration displays even higher impedance values in comparison to the lowest concentration, which can be attributed to the higher degree of agglomeration, which is inevitable due to the large quantity of nanoparticles present in the dispersion.

Thus, the lowest impedance modulus is obtained for the 0.10 wt.% dispersion and 65 kJ ultrasonic energy. Slightly lower, but similar values are obtained for the 100-kJ ultrasonic energy at the same concentration, but 65 kJ was chosen for the following reasons: (1) it would be more time-efficient and practical, (2) it allows for clearer comparison to the other nanostructures’ dispersions, for which a different concentration was found to be the optimal (0.15 wt.%). Although, the MWCNTsOSO_3_H dispersion was then produced at different concentration, the ultrasonic energy amount was the same as in the other CBNs.

The Bode impedance plots in Figure 6 refer to the four MWCNTsCOOH aqueous dispersions and present the impedance vector against test frequency for different accumulated ultrasonication energy values. At the lowest concentration, impedance decreases as ultrasonic energy increases. This is logical since the agglomerates break down with an increase in ultrasonic energy, and therefore more uniform networks are formed for electrical current to pass. However, the nanoparticles are not dense enough and therefore even at higher ultrasonic energies, the impedance levels are high. At all higher concentrations, the impedance decreases as ultrasonic energy increases up to a certain energy level. After that point, impedance increases with an increase in ultrasonic energy. Again, the application of ultrasonic energy leads to a more uniform dispersion of the specific carbon nanostructure.

**Figure 7:**
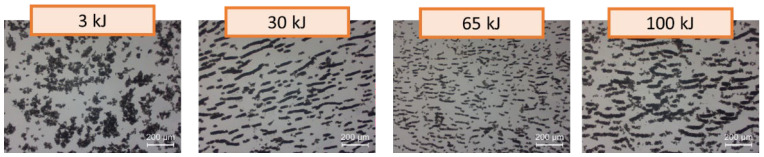
Optical microscopy images for MWCNTsCOOH at different ultrasonic energy levels (0.15 wt.% concentration).

The impedance test results were also supported by several analytical methods. Figure 7 displays characteristic optical microscopy images for the carboxylated MWCNTs at different ultrasonic energy levels, all referring to the same concentration (0.15 wt.%). Large agglomerates can be noticed for the very low ultrasonic energy levels. Those agglomerates start to break until ultrasonic energy reaches a certain amount, approximately around 65 kJ. Higher levels of ultrasonic energy affect the surface functionalization of the nanostructures; breaking of the surface-attached functional groups is assumed that finally results into re-agglomeration, e.g., at 100 kJ ultrasonic energy.

As noticed in relevant research articles, e.g., [25,26], graphene oxide dispersions exhibit lower impedance values, since it contains many different conductive functional groups. In contrast, rGO dispersions exhibit the highest impedance values in comparison with the other nanostructures. More specifically, impedance values are more than 10 times higher in lower ultrasonic energy values. This is attributed to the absence of conductive functional groups. Comparing GO and functionalized CNTs, it is well known that there is a stronger hydrogen bonding between the GO interlayer induced by a large amount of oxygen functional groups and flexible two-dimensional morphology with a large surface area.

### 3.2. Electrical Resistance Results

Building materials, not only restoration binders, do not possess electrical properties and can be considered by no means as conductive. On the other hand, graphene and CNTs possess extraordinary electrical properties, more advanced in comparison to graphite and carbon black [30]. In some cases of functionalized nanomaterials, also possess enhanced electrical properties, though the covalent attachment of functional groups with higher electron-withdrawing affinity leads more in semiconducting character [30]. Thus, it was expected that the incorporation of CBNs would decrease the electrical resistance of the composite pastes.

**Figure 8:**
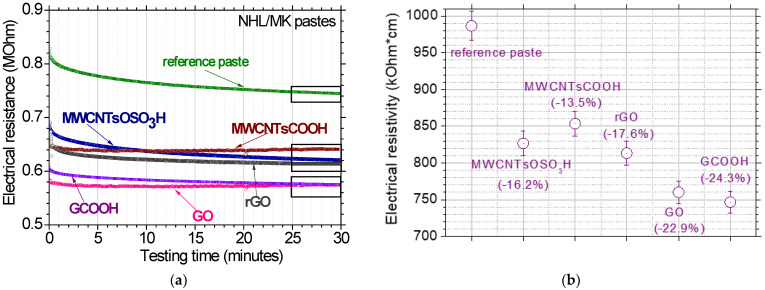
(**a**) Electrical resistance testing results for all investigated pastes and (**b**) electrical resistivity of the respective nano reinforced restoration pastes.

Indeed, the test results of the present investigation confirmed the hypothesis, as seen in Figure 8. More specifically, the reference paste exhibited electrical resistance ~0.74 MOhm. The incorporation of modified CNTs and rGO led to a 13% to 17% reduction in the electrical resistance, while the equivalent reduction in the case of GO and GCOOH was approximate by 23%. The electrical resistance was converted to electrical resistivity based on Ohm’s Law, so that the values can be exploited at specimens with different geometrical characteristics. The respective values for the electrical resistivity can be seen in the right diagram of Figure 8. It is evident that the percentage reduction of the electrical resistance is equal to the percentage reduction of the electrical resistivity.

In the case of electrical properties of the composite materials, the literature review revealed that the test results are more consistent. For instance, Cerro-Prada et al. [31] found that electrical resistivity of mortars with 0.01 and 0.015 wt.% MWCNTs resulted up to 10% decrease at both 28- and 90-days curing. Jang et al. [32] found that the electrical conductivity of 0.5 wt.% MWCNT/cement pastes with/without surfactant was about 50% and 20% higher, respectively, than the electrical conductivity of plain cement paste. Najafishad et al. [33] found that the incorporation of 0.15 wt.% GO in cement leads to 70% decrease in electrical resistance, while the same concentration of CNTs leads to 78% respective decrease. Zhang et al. [34] examined the effect of rGO (up to 4 wt.%) in cementitious matrix. They found that electrical resistivity decreases with increasing concentration of rGO, reaching a plateau at 2%, where the electrical resistivity was reduced by 40%.

However, a few studies report an adverse effect with the incorporation of carbon nanostructures. For instance, Kaur and Kothiyal [35] found that resistivity increased with the addition of GO. In addition, Kim et al. [36] found that CNTs had an insignificant effect on the electrical resistance of the cement composites. Nonetheless, when a pozzolanic material was added, the resistivity dropped significantly [36]. This can be linked to our case, since metakaolin was added as pozzolanic material. Li et al. [37] reported that this effect is attributed to the nucleation on GO during the acceleration stage of the cement hydration process. This can happen when GO is added at low concentrations. At higher concentrations, the electrical resistivity decreases, since the number of nanoparticles is high enough to cause ion diffusion due the carboxylic acid groups [37].

### 3.3. Mechanical Testing Results

**Figure 9:**
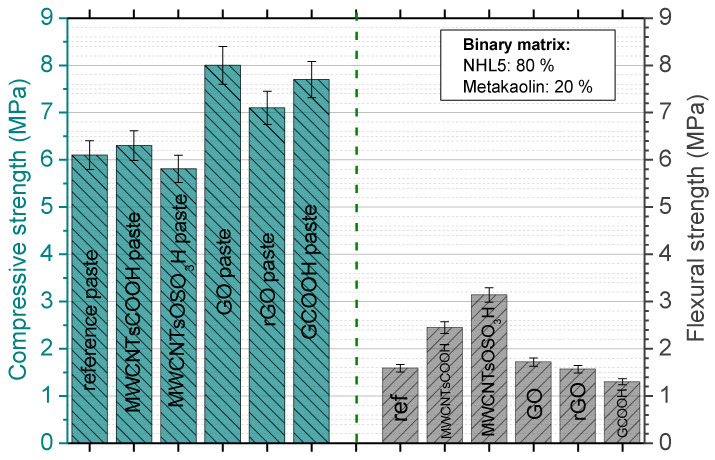
Mechanical test results of compressive and flexural strengths of the NHL/MK pastes reinforced with different type of carbon nanostructures (concentrations can be found in Table 2).

Figure 9 presents the mechanical test results obtained from the compressive and flexural tests. The reference pastes displayed 6.1 MPa compressive strength and 1.6 MPa flexural strength, respectively. Regarding the effect of MWCNTs reinforcement (in both MWCNTs investigated), it was found that their addition in compressive strength is minor, while the effect in flexural strength is very significant. More specifically, compressive strength of MWCNTsCOOH specimens was only ~2% higher and compressive strength of MWCNTsOSO3H specimens was approximate 6% lower than the respective reference paste values. In both cases, the divergence lies withing the standard deviation range. On the other hand, the incorporation of MWCNTsCOOH and MWCNTsOSO_3_H leads to a 60% and 110% increase in compressive strength, respectively. This means that in the case of sulfonated carbon nanotubes, flexural strength is more than doubled.

Worth noticing is that the literature review reveals a variety of test results in the subject, e.g., [38]. For instance, Kaur and Kothiyal [35] investigated the effect of functionalized MWCNTs in cement paste at different concentrations and found that the average improvement in compressive strength of all incorporation ratios of MWCNTs was 10% at 28 days of age. Although, it is expected that the incorporation of CBNs increases the mechanical strength when added up to a specific concentration (usually higher than 1.0%), Sindu and Sasmal [39] found that after the incorporation of 0.08 wt.%, the compressive strength decreases for all studied cases. The highest reduction in the compressive strength was observed with 0.3 wt.% of MWCNTs incorporation and was approximately by 94%.

“Controversial” results are also reported concerning the effect of MWCNTs on flexural strength. For instance, Konsta-Gdoutos et al. [40] found that the incorporation of MWCNTs at 0.1 wt.% increased the flexural strength of cement mortars by 119%, while other studies reported decrease in flexural strength with the incorporation of the same amount of CNTs [38]. In all cases, the positive effects of the incorporation of CNTs on the mechanical properties of the composites were attributed to the dispersion technique’s effectiveness in yielding individually dispersed MWCNTs, which included a surfactant, ultrasonication and/or functionalization. In contrast, decrease in mechanical strength is linked to bad dispersion quality or high-water absorption by the surfactants [38].

Hu et al. [41] found that the addition of MWCNTs-COOH increased the compressive strength by approximately 5%, while other mechanical properties were affected more significantly, which is totally in accordance with the present study. Nevertheless, it must be noted that the investigated pastes of the present study are compared with cement-based nanocomposites due to the literature gap in the restoration materials. It is assumed that similar mechanisms are applied in the present case of other hydraulic binders. The dispersion quality is still a crucial factor, maybe the most critical one, to determine the final properties of the composite material. Uniform dispersion leads to decrease in pore size and good bonding between MWCNTs and the matrix [41].

As far as the graphene nanostructures are concerned, the incorporation of modified graphene structures leads to an enhancement of compressive strength. More specifically, the addition of GO, rGO and GCOOH enhances the compressive strength by 29%, 15% and 24%, respectively. The incorporation of GO increases flexural strength about 7%, while the incorporation of GCOOH reduces flexural strength by about 15%. The addition of rGO led to 2% reduced flexural strength, a value that lies within the ranges of standard deviation, and thus the difference can be considered as negligible.

As in the case of MWCNTs, literature review in graphene also reveals controversial results regarding the effect of graphene-based structures in cementitious materials [42]. Sheikh et al. [43] reported that the variety of results observed in different studies lies on the following factors: synthesis process, functional group ratios, oxidation content, lateral sheet sizes and number of stacked sheets per GO agglomerate. Even minor changes in these factors lead to different results, therefore it is very difficult to control the outcome and ensure replicability [43].

When GO nanoparticles are efficiently dispersed in the cementitious matrix, they increase coherence through the bonding with C-S-H phase and delay propagation of microcracks. This leads to enhancement of mechanical properties [42]. Efficient dispersion is achieved due to the presence of hydroxyl (OH-), carboxyl (-COOH), and carbonyl (=O) functional groups, that are soluble in water. In addition, the acidic functional groups react with Ca(OH)_2_ and C-S-H, ensuring bonding between the nanoparticles and cement phase. However, high concentrations of GO (thus many particles) form agglomerates and van der Waal forces cannot be overcome. This can cause defects in the cement matrix and mechanical properties may even be reduced [42]. All the examined graphene nanostructures contain oxygen functional groups. GO and GCOOH contain more when compared to rGO and this can explain why rGO paste displayed the least increase in compressive strength.

Although the hardening and hydration mechanisms are not the same between NHL and cement, similar principles can be applied in the case of their connection with CBNs. For instance, hydration of NHL leads to the formation of portlandite and Ca(OH)_2_, as in the case of cement [44]. Zhang et al. [45] studied the properties of two different natural hydraulic limes, cement-aerial lime, and slag-aerial lime mixtures. Regarding the hydration reactions, they occur in the early stages of paste hardening in the case of cement-lime and NHL mixtures, while the pozzolanic reaction also achieves high degree in the early hardening. Moreover, the carbonation reaction process of the three kinds of lime pastes continues to the later period of the hardening. The hydration process follows a similar path and thus, the link among the nanoparticles and the binder matrix takes place in the equivalent stages.

### 3.4. Piezoresistivity under Cyclic Loading

**Figure 10:**
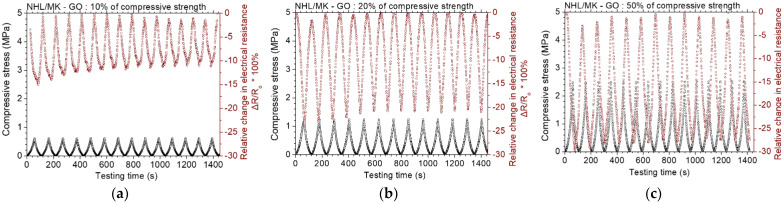
Cyclic, piezoresistive response of GO reinforced binary paste at constant amplitude level of (**a**) 10%, (**b**) 20% and (**c**) 50% of the compressive strength of the paste.

The previous test results showed that all the incorporated carbon nanostructures decreased the electrical resistivity of the binder materials significantly. As a next step, the five different investigated pastes were examined regarding their piezoresistive characteristics. Figure 10 displays the test results of the piezoresistive experiments for the NHL/MK pastes with incorporated GO. At cyclic compressive loadings up to 10% of the compressive strength, the maximum change in electrical resistance was approximate 10%. At cyclic loadings up to 20% of the compressive strength, the maximum change in electrical resistance is about 20%, which was highly appreciated. Finally, at loadings up to 50% of the compressive strength, the maximum change in electrical resistance is approximate 28%. Guo et al. [15] studied the piezoresistive characteristics of cement/GO composites and reported that a conductive network with tunneling effect and contacting conduction is effectively formed by GO sheets in the cement matrix. Under cyclic loading, the distances between adjacent GO sheets change together with the variations of strain load and thus lead to synchronous piezoresistive responses.

**Figure 11:**
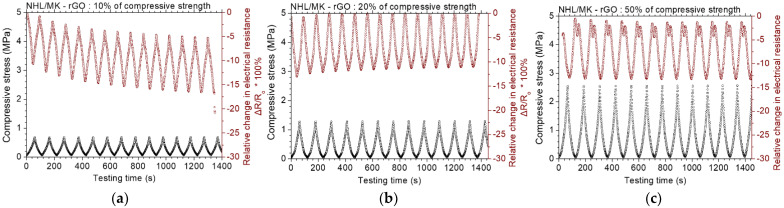
Cyclic, piezoresistive response of rGO reinforced binary paste at constant amplitude level of (**a**) 10%, (**b**) 20% and (**c**) 50% of the compressive strength of the paste.

**Figure 12:**
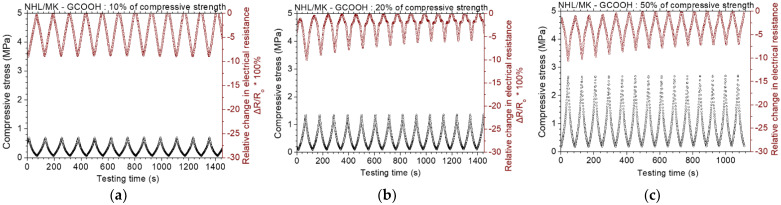
Cyclic, piezoresistive response of GCOOH reinforced binary paste at constant amplitude level of (**a**) 10%, (**b**) 20% and (**c**) 50% of the compressive strength of the paste.

Figure 11 displays the results of the piezoresistive experiments for the NHL/MK pastes with incorporated rGO. At loadings up to 10% of the compressive strength, the maximum change in electrical resistance is about 10%. At loadings up to 20% and 50% of the compressive strength, the piezoresistive response remains almost unaltered. It is noted that at the lower loading (10%), the electrical resistance change keeps a constant change of 10%, but the absolute value of the electrical resistance decreases. Zhang et al. [34] reported similar behavior of rGO-cement mortars with 0.5 wt.% rGO. They found out that the piezoresistive response stabilizes at higher concentrations of rGO [34]. Figure 12 displays the results of the piezoresistive experiments for the NHL/MK pastes with incorporated GCOOH. In contrast with the two former graphene nanostructures, GCOOH pastes do not show enhanced piezoresistive response, but the percentage of resistance change remains practically constant for all loading levels. GCOOH displays a different behavior in the case of blended lime-cement binders, with outstanding piezoresistive response, as shown in previous work of the authors [26].

**Figure 13:**
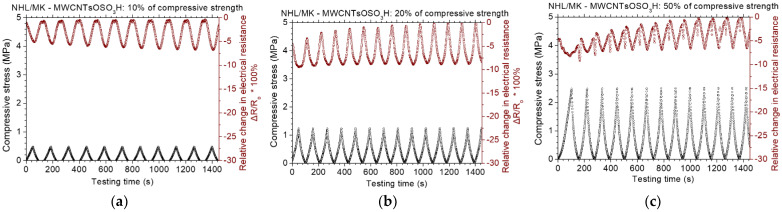
Cyclic, piezoresistive response of MWCNTsOSO_3_H reinforced binary paste at constant amplitude level of (**a**) 10%, (**b**) 20% and (**c**) 50% of the compressive strength of the paste.

**Figure 14:**
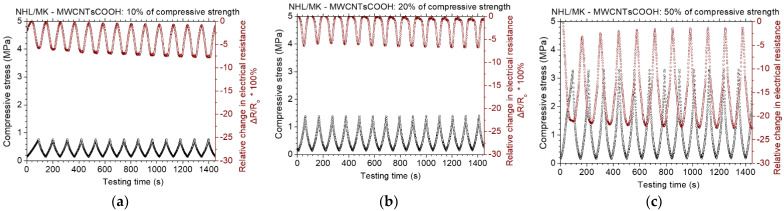
Cyclic, piezoresistive response of MWCNTsCOOH reinforced binary paste at constant amplitude level of (**a**) 10%, (**b**) 20% and (**c**) 50% of the compressive strength of the paste.

Figure 13 displays the results of the piezoresistive experiments for the NHL/MK pastes with incorporated sulfonated MWCNTs. At loadings up to 10% of compressive strength, the maximum change in electrical resistance is approximate 5%. At loadings up to 20% of the compressive strength, the maximum change in electrical resistance is about 10%, while at loadings up to 50% of the compressive strength, the piezoresistive response is not very stable. This means that the piezoresistive capacity of MWCNTsOSO_3_H reinforced pastes is reduced at high loading levels. The piezoresistive results for the NHL/MK pastes with incorporated carboxylated MWCNTs can be found in Figure 14. At loadings up to 10% and 20% of the compressive strength, the maximum change in electrical resistance is about 7%. At loadings up to 50% of the compressive strength, the maximum change in electrical resistance is about 22%. In comparison to the sulfonated MWCNTs, the behavior of MWCNTsCOOH pastes is deemed better. At lower loadings, it is not that important to show significant electrical resistance change. However, the behavior under extensive loading levels is very important in the case of restoration applications.

**Figure 15:**
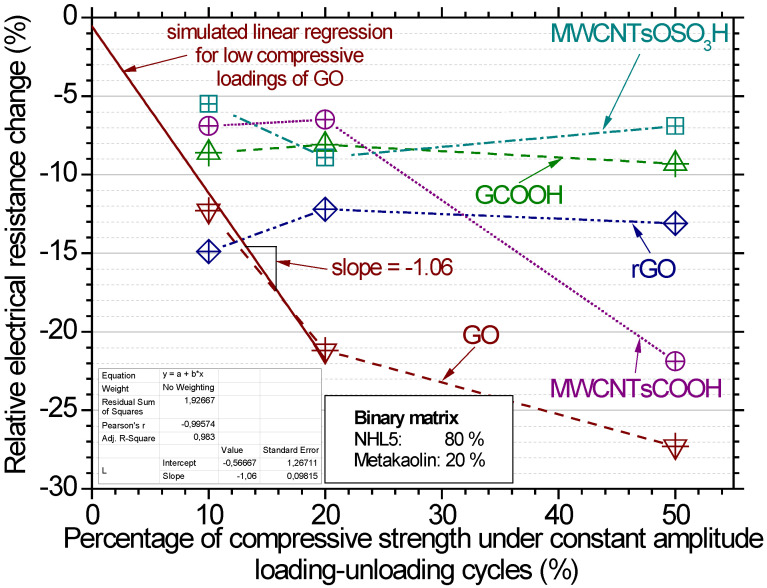
Average relative resistance change (%) for all investigated carbon nanostructures reinforced pastes for the different mechanical loading levels under compression.

Figure 15 displays the average relative electrical resistance change for all investigated pastes at the three different cycling loading levels. At the lowest lever, the greatest response is noted for the rGO pastes, where an approximate 15% drop is noticed. At the intermediate and highest loading level, the greatest response is noted for the GO composite pastes and approximately 21% and 27% drop, respectively. The GCOOH-, MWCNTsCOOH- and MWCNTsOSO_3_H-pastes show similar response at the 10% and 20% loading levels, ranging from 5 to 10% drop in electrical resistance. The worst piezoresistive response is noticed for the carboxylated graphene composite pastes and the sulfonated CNTs pastes. Regarding the MWCNTsCOOH paste, it is observed that at 50% loading level the piezoresistive response is very significant, while at the lower loading levels the response is around 7%. This behavior poses the specific nanostructure as a good candidate to be used for restoration of Monuments. In general, based on the piezoresistive response, the ranking of the involved composite pastes regarding their sensing ability in terms of electrical resistance change with varying compressive stress is the following:

GO > MWCNTsCOOH > rGO > GCOOH ≈ MWCNTsOSO_3_H.

The most promising nano reinforcement for structural health monitoring according to the authors seems to be the GO, as can be seen in Figure 15. This can be supported by the almost linear increase of fractional resistance change with the increase of compressive cyclic loading for the GO up to the 20% of monotonic compressive strength. Hence, the mechanical load is analogous to the piezoresistive response of the paste, while this was not noticed for no other investigated paste. The inverse analogous response was fitted by linear regression and the slope of the equation was found to be equal to 1.09 (more details on the equation can be found in the insert of Figure 15).

The results reveal that both, CNTs and GO can improve the piezoresistive properties of composites. GO’s performance can be attributed to the strong hydrogen bonds between the GO interlayer induced by a large amount of oxygen functional groups and flexible two-dimensional morphology with a large surface area [46]. The differences in the examined properties can be attributed to the different structure of CNTs and graphene. Since CNTs is a one-dimensional wire-like material, the CNT addition is beneficial for increasing the contact area between the nanomaterial, creating nanosized mechanical interlocking [47]. This is also the reason why the incorporation of CNTs leads to such significant enhancement in flexural strength and not compressive strength. In contrast, GO is a two-dimensional platelet material with higher surface free energy than CNTs [47]. When GO particles contact the matrix, an interfacial mechanical interlocking and chemical bonding is created. The platy structure of GO makes the composite material more resilient to compressive loadings.

## 4. Conclusions

In the present study, natural hydraulic lime (NHL)/metakaolin (MK) pastes with incorporated functionalized carbon-based nanomaterials were produced and their mechanical, electrical and piezoresistive properties were examined. Although extensive studies can be found in literature referring to the incorporation of carbon-based nanomaterials (CBNs) in cement matrices, the present study investigates for the first time the piezoresistive characteristics of functionalized CBNs in NHL restoration pastes. It is expected that the present study will lay the groundwork for the development of self-sensing restoration materials. Five different functionalized CBNs were incorporated in the blended pastes and the outcome can be summarized as follows:

- GO pastes increased compressive strength by 29% and flexural strength by 7%, while the electrical resistivity of the GO pastes was about 23% lower than this of the reference pastes. These samples showed evident piezoresistive response, and an almost linear increase of electrical resistance change was noticed with increasing cyclic loading level. This seems to be the promising paste as it can be used for quantitative purposes of damage identification of the paste.
- rGO pastes increased compressive strength by 15% and flexural strength remained almost constant, while the electrical resistivity of the rGO pastes was about 18% lower than this of the reference pastes. rGO pastes also showed piezoresistive response, nevertheless at mechanical loadings up to 20% and 50% of the compressive strength, the respective electrical response was the same, thus assuming that can be used only for qualitative damage identification purposes.
- GCOOH pastes increased compressive strength by 24%, while flexural strength was reduced by 15%. The electrical resistivity of the GCOOH pastes was 24% lower, when compared to the reference samples. In comparison to the other graphene structures, GCOOH performed poorly in the piezoresistive experiments.
- Compressive strength of MWCNTsCOOH pastes remained unchanged, while the flexural strength was increased by 60%. The electrical resistivity of MWCNTsCOOH pastes was about 14% lower than the resistivity of the reference paste. Regarding the piezoresistive response, at lower loadings it remained unchanged, but at 50% of compressive strength it was the same as in the case of GO.
- Compressive strength of MWCNTsOSO_3_H pastes did not change significantly, while the flexural strength was doubled. The electrical resistivity of those pastes was 16% lower compared to the reference samples. In contrast to MWCNTsCOOH, the piezoresistive response of sulfonated CNTs pastes was better at lower loadings. At the highest loading, the performance was poor.

The following ranking of the pastes is suggested based on the piezoresistive results:

GO > MWCNTsCOOH > rGO > GCOOH ≈ MWCNTsOSO_3_H.

In contrast with the case of cement, where durability and increase in mechanical properties is very important, in the case of restoration binders a very high increase in mechanical properties is usually undesirable; this is the reason why the ranking is based on the piezoresistive response only.

## Figures and Tables

**Table 1:** Specifications of binder materials.

Natural Hydraulic Lime (NHL5)	Metakaolin
Free lime: > 20%	Al_2_O_3_ + SiO_2_: 97%
SO_3_: > 2%	Chlorides: < 0.01%
Particle size 90 µm: ≤ 15%	Free lime: < 0.01%
Particle size 200 µm: ≤ 2%	Fe_2_O_3_ (%): < 0.6%

**Table 2:** Composition of the produced pastes.

No	Paste Name	Binder Composition	Nanomaterial Concentration (wt.% of the Binder)
1	Reference paste	80 wt.% NLH5/20 wt.% MK	0.00 (0 kJ ultrasonic energy)
2	GO-paste	80 wt.% NLH5/20 wt.% MK	0.15 (65 kJ ultrasonic energy)
3	GCOOH-paste	80 wt.% NLH5/20 wt.% MK	0.15 (65 kJ ultrasonic energy)
4	rGO-paste	80 wt.% NLH5/20 wt.% MK	0.15 (65 kJ ultrasonic energy)
5	MWCNTsCOOH-paste	80 wt.% NLH5/20 wt.% MK	0.15 (65 kJ ultrasonic energy)
6	MWCNTsOSO_3_H-paste	80 wt.% NLH5/20 wt.% MK	0.10 (65 kJ ultrasonic energy)

## Data Availability

Not applicable.
